# EspM2 is a RhoA guanine nucleotide exchange factor

**DOI:** 10.1111/j.1462-5822.2009.01423.x

**Published:** 2010-02-05

**Authors:** Ana Arbeloa, James Garnett, James Lillington, Richard R Bulgin, Cedric N Berger, Susan M Lea, Steve Matthews, Gad Frankel

**Affiliations:** 1 Centre for Molecular Microbiology and Infection, Imperial College LondonLondon, UK; 2 Division of Molecular Biosciences, Imperial College LondonLondon, UK; 3 Sir William Dunn School of Pathology, University of OxfordOxford, UK; 4 Inorganic Chemistry Laboratory, University of OxfordOxford, UK

## Abstract

We investigated how the type III secretion system WxxxE effectors EspM2 of enterohaemorrhagic *Escherichia coli*, which triggers stress fibre formation, and SifA of *Salmonella enterica* serovar Typhimurium, which is involved in intracellular survival, modulate Rho GTPases. We identified a direct interaction between EspM2 or SifA and nucleotide-free RhoA. Nuclear Magnetic Resonance Spectroscopy revealed that EspM2 has a similar fold to SifA and the guanine nucleotide exchange factor (GEF) effector SopE. EspM2 induced nucleotide exchange in RhoA but not in Rac1 or H-Ras, while SifA induced nucleotide exchange in none of them. Mutating W70 of the WxxxE motif or L118 and I127 residues, which surround the catalytic loop, affected the stability of EspM2. Substitution of Q124, located within the catalytic loop of EspM2, with alanine, greatly attenuated the RhoA GEF activity *in vitro* and the ability of EspM2 to induce stress fibres upon ectopic expression. These results suggest that binding of SifA to RhoA does not trigger nucleotide exchange while EspM2 is a unique Rho GTPase GEF.

## Introduction

Pathogenic bacteria use a variety of strategies to subvert cellular and immunological functions to facilitate colonization, multiplication and survival within the host. Several Gram-negative pathogens, e.g. *Salmonella enterica*, *Shigella* spp. and enteropathogenic and enterohaemorragic *Escherichia coli* (EPEC and EHEC), encode a type III secretion system (T3SS) which is central for their infection strategy. T3SS are molecular syringes that allow translocation of effector proteins directly from the bacteria to the cytoplasm of the host cell. Once translocated the effectors subvert cellular processes to facilitate the particular infection style of the pathogen (Mota and Cornelis, 2005). To this end bacterial T3SS effectors often display sequence, structural or functional similarities to eukaryotic proteins.

Rho GTPases react to a range of intrinsic and extrinsic stimuli in order to regulate a plethora of host cell signalling networks most notably those involved in remodelling the eukaryotic actin cytoskeleton and, as such, are prominent targets of T3SS effectors (Finlay, 2005). RhoA, Cdc42 and Rac1, the most studied Rho GTPases, induce formation of stress fibres, filopodia and lamellipodia respectively (Jaffe and Hall, 2005). To exert their control on these cellular processes Rho GTPases act as molecular switches cycling between GTP-bound ‘on’ and GDP-bound ‘off’ conformations. Rho GTPases have well-defined nucleotide and magnesium binding pocket, constituted mainly by two polypeptides called Switch I and II and by the phosphate-binding loop or P-loop. Mg^2+^ ions are required for high-affinity binding of guanine nucleotides to Rho GTPases. The Switch I and II regions define the major conformational differences between the GDP and GTP bound forms; only the GTP-bound conformation allows interactions of the Rho GTPases with their downstream effectors. The activation state of Rho GTPases is modulated by three main categories of regulatory proteins: (i) guanine nucleotide dissociation inhibitors (GDI) that sequester GTPases in the cytosol in a GDP-bound state, (ii) guanine nucleotide exchange factors (GEFs) that catalyse the GDP/GTP exchange, and (iii) GTPase activating proteins (GAPs) that inactivate the Rho GTPases by stimulating their intrinsic GTPase activity.

Type III secretion system effectors use different mechanisms to subvert Rho GTPases. For example, EPEC and EHEC EspG and EspG2 indirectly activate RhoA by disrupting microtubules, which leads to liberation of a RhoA-specific GEF, GEF-H1 (Matsuzawa *et al.*, 2004). Other T3SS effectors modulate Rho GTPase directly as they function as either GEFs or GAPs. For example, the *Salmonella* effector SopE directly activates Rac1 and Cdc42 leading to lamellipodia formation and promoting bacterial invasion into non-phagocytic cells (Hardt *et al.*, 1998). In contrast, *Salmonella* uses the GAP T3SS effector SptP to stimulate the intrinsic Rho GTPase activity to restore cell architecture following bacterial internalization (Fu and Galan, 1999). SopE and SptP homologues have been identified in different species including *Burkholderia pseudomallei* (BopE), *Yersinia pseudotuberculosis* (YopE) and *Pseudomonas aeruginosa* (ExoS) (Goehring *et al.*, 1999; Von Pawel-Rammingen *et al.*, 2000; Stevens *et al.*, 2003). By modulating the actin cytoskeleton via Rho GTPases, YopE and ExoS inhibit bacterial uptake by macrophages (Black and Bliska, 2000; Deng and Barbieri, 2008).

Based on a conserved motif comprising an invariant tryptophan (W) and a glutamic acid (E) separated by three variable amino acids, Alto *et al.* (2006) grouped together a number of known T3SS effectors from *Shigella* (IpgB1 and IpgB2), *Salmonella* (SifA and SifB) and EPEC and EHEC (Map) and termed them WxxxE effectors. Recently, we identified new WxxxE effectors encoded by EPEC and EHEC, EspM (Tobe *et al.*, 2006) and EspT (Bulgin *et al.*, 2009). Ectopic expression of Map leads to filopodia formation (Alto *et al.*, 2006), IpgB2 and EspM trigger stress fibres (Alto *et al.*, 2006; Arbeloa *et al.*, 2008) and IpgB1 and EspT induce membrane ruffles and lamellipodia (Alto *et al.*, 2006; Bulgin *et al.*, 2009). These phenotypes are typically associated with activated Cdc42, RhoA and Rac1 (Hall *et al.*, 1993) respectively.

Alto *et al.* suggested that the WxxxE effectors, which play important roles in cell invasion (IpgB proteins) and intracellular survival (SifA), mimic the function of Rho GTPases. Handa *et al.* (2007) subsequently demonstrated that IpgB1 stimulates formation of membrane ruffles by activating Rac1 through recruitment of the Rac1-specific ELMO–Dock180 GEF complex. Moreover, the structure of SifA in complex with the PH domain of SKIP has shown that its C-terminus domain, which includes the WxxxE motif, adopts a fold similar to SopE (Ohlson *et al.*, 2008). However, neither direct binding to the Rho GTPases nor GEF activity was detected in this study. Furthermore, we have recently reported that Map (Berger *et al.*, 2009), EspM (Arbeloa *et al.*, 2008) and EspT (Bulgin *et al.*, 2009) activate the Rho GTPases Cdc42, RhoA and Rac1. The aim of this study was to determine the mechanism through which EspM2 activates RhoA.

## Results

### EspM2 binds RhoA

We used His-EspM2 to investigate if activation of RhoA involves a direct interaction. Although highly soluble, His-EspM2 was unstable and appeared in two distinct bands corresponding to the full-length effector and a spontaneous degradation product; N-terminal sequencing revealed that the latter corresponds to EspM2 lacking the first 28 amino acids (EspM2^29–196^) (Appendix S1 and Fig. S1). Ectopic expression of EspM2^29–196^ in Swiss 3T3 cells resulted in stress fibre formation at the same level as full-length EspM2 (Fig. S2), confirming that it retained full biological activity. Consequently we have used EspM2^29–196^ throughout this study.

**Fig. 1:**
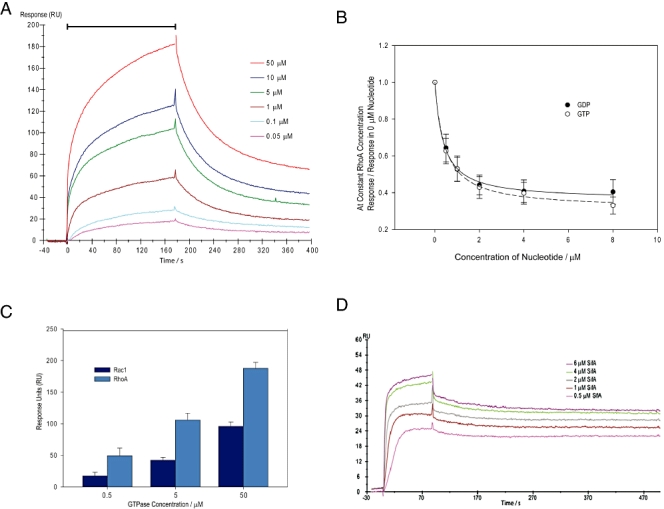
RhoA binds directly to EspM2 and SifA. A. Surface plasmon resonance showing concentration-dependent binding of RhoA to EspM2^29–196^. Concentrations of RhoA varying from 0.05 µM to 50 µM were flowed at 50 µl min^−1^ (duration indicated by black bar) over a CM5 sensor chip with EspM2^29–196^ covalently bound to the surface. Control substituted signals are shown. B. Inhibition of EspM2^29–196^–RhoA interaction by GDP or GTP. For RhoA (4 µM) flowing over an EspM2^29–196^-bound surface in buffer containing GDP/GTP, the response maxima relative to response maxima in the absence of nucleotide are plotted with respect to GDP/GTP concentration. C. Binding of EspM2^29–196^ to RhoA and Rac1. Averaged response maxima for three representative concentrations compare the strength of EspM2 binding with the two GTPases. D. Surface plasmon resonance demonstrates RhoA concentration dependence for binding SifA. Concentrations of SifA varying from 0.5 µM to 6 µM were flowed at 50 µl min^−1^ over a CM5 sensor chip with RhoA covalently bound to the surface.

Surface plasmon resonance was used to probe the interaction between His-EspM2^29–196^ and His-RhoA. This technique allows binding to be observed in real time by the change in mass over the derivatized surface of a sensor chip. RhoA was flowed (sequential injections ranging from 0.05 µM to 50 µM) over an EspM2^29–196^-bound surface and displayed an increased rate of binding with concentration confirming a specific interaction (Fig. 1A). Free GDP or GTP added to the running buffer inhibited formation of an EspM2^29–196^–RhoA complex in a nucleotide concentration-dependent manner (Fig. 1B). GDP and GTP have the same effect as one another (within experimental error), reducing the binding affinity of EspM2^29–196^ to RhoA by up to 67% in the nucleotide concentration range 0.5–8 µM.

As a control, Rac1 was flowed over the same EspM2^29–196^ surface and for any given GTPase concentration showed substantially less binding (Fig. 1C), confirming the specificity of EspM2^29–196^ for RhoA over other GTPases. As recent data suggested that SifA might bind RhoA (Ohlson *et al.*, 2008), their direct interaction was also tested. We found that His-SifA and His–RhoA bound in a concentration-dependent manner (Fig. 1D), and was inhibited by free GDP or GTP (data not shown). Although high RhoA concentrations (µM range) were required to see an interaction, EspM2^29–196^–RhoA binding was found to be long-lived. The interaction response was followed for 1000 s after each injection, by which time the rate of dissociation appeared to have fallen to zero in an incompletely dissociated state. Dissociation of the pre-formed EspM2^29–196^–RhoA, as well as SifA–RhoA, complexes could not be brought about by addition of 500 µM free GDP in running buffer (in the presence or absence of 5 mM MgCl_2_, data not shown) in contrast to the rapid dissociation of SopE–Cdc42 reported by Rudolph *et al.* (1999). Dissociation of EspM2^29–196^–RhoA proved to be more difficult than that of SifA–RhoA, requiring high pH conditions (25 mM NaOH, pH 12.4), where high ionic strength (1 M NaCl) would suffice for the latter to bring the response back to the pre-experiment level.

### EspM2 stimulates guanine nucleotide exchange in RhoA

**Fig. 2:**
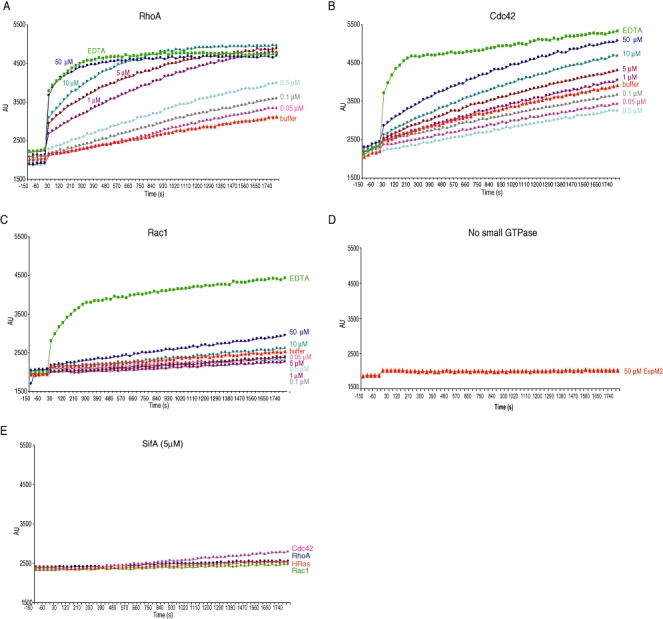
EspM2 is a RhoA GEF. A. EspM2^29–196^ mediates loading of mant-GTP into RhoA. mant-GTP (0.5 µM) was incubated with 2 µM RhoA in presence of 250 mM EDTA (squares), or 0.05–50 µM EspM2^29–196^ (circles) or in presence of buffer only (triangles). The insertion of the mant-GTP into the nucleotide binding pocket of RhoA in presence of EspM2^29–196^ detected by an increase in the fluorescent emission was found to be concentration dependent. B. EspM2^29–196^ weakly induces loading of mant-GTP into Cdc42. mant-GTP (0.5 µM) was incubated with 2 µM Cdc42 in presence of 250 mM EDTA (squares), or 0.05–50 µM EspM2^29–196^ (circles) or in presence of buffer only (triangles). Slow loading of mant-GTP into Cdc42 was only detected when high concentrations of EspM2^29–196^ were added. C. EspM2^29–196^ does not induce nucleotide exchange for Rac1. mant-GTP (0.5 µM) was incubated with 2 µM Rac1 in presence of 250 mM EDTA (squares), or 0.05–50 µM EspM2^29–196^ (circles) or in presence of buffer only (triangles). No efficient loading of man-GTP into Rac1 was observed after incubation with up to 50 µM EspM2^29–196^. D. Incubation of 50 µM EspM2^29–196^ in the exchange buffer alone did not change the fluorescence intensity. E. SifA does not induce nucleotide exchange in RhoA, Cdc42, Rac1 or H-Ras. mant-GTP (0.5 µM) was incubated with 2 µM RhoA (blue circles), Cdc42 (pink circles), Rac1 (green circles) or H-Ras (orange circles) in presence of 5 µM SifA. No loading of man-GTP into any of the small GTPases tested was observed in these conditions. Results shown are the average of three independent experiments.

We next investigated the ability of EspM2^29–196^ to stimulate guanine nucleotide exchange in RhoA and other GTPases *in vitro* using a RhoGEF exchange assay. This spectroscopic assay measures fluorescent emission upon insertion of *N*-methylanthraniloyl(mant)-GTP into the nucleotide binding pocket of the GTPases (Rossman *et al.*, 2002). As shown in Fig. 2 inclusion of 250 mM EDTA (positive control) in the assay induced efficient nucleotide exchange in RhoA, Cdc42, Rac1 and H-Ras, indicating that these proteins were biologically functional, while a slow intrinsic nucleotide exchange activity was detected in the presence of buffer alone (negative control). The fluorescence intensity rose dramatically and in a concentration-dependent manner when increasing amounts of purified EspM2^29–196^ were added to RhoA (Fig. 2A). In contrast, EspM2^29–196^ showed a weak exchange activity in Cdc42 (Fig. 2B), whereas it had no effect on Rac-1 and H-Ras (Fig. 2C and data not shown). No change in fluorescence emission was detected when EspM2 was incubated in the absence of any of the small GTPases (Fig. 2D). Testing the GEF activity of SifA revealed that it did not stimulate nucleotide exchange in any of the tested Rho GTPases or H-Ras (Fig. 2E). These results demonstrate that EspM2^29–196^ is a specific RhoA GEF, and that although SifA can bind RhoA it cannot induce nucleotide exchange.

### Mapping the EspM2 interface of the EspM2–RhoA complex

**Fig. 3:**
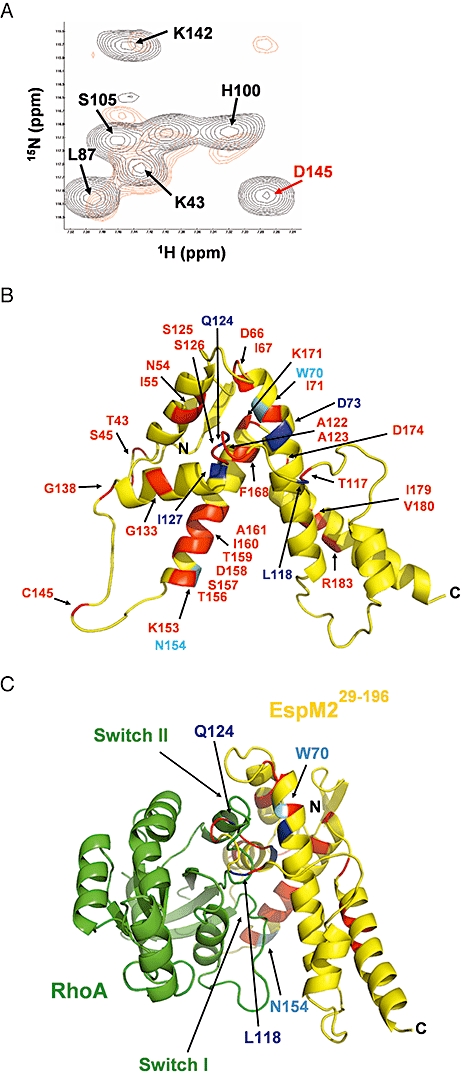
Model of EspM2^29–196^ and the EspM2^29–196^–RhoA complex. A. ^1^H ^15^N TROSY-HSQC titration of RhoA against EspM2^29–196^. Black peaks show no addition of RhoA while red peaks show fourfold molar excess of RhoA. Chemical shift changes were deemed significant if the peak intensity was reduced by more than 80% (i.e. D145). B. Homology model of EspM2^29–196^ created with SWISS-MODEL (Arnold *et al.*, 2006). Data from the RhoA NMR titration (red) and alanine mutations (blue) have been mapped onto the model. Those residues with chemical shift changes in the NMR titrations and which have also been mutated to alanines are coloured cyan. C. Model of the EspM2^29–196^–RhoA complex created by superimposing the EspM2^29–196^ model and the crystal structure of RhoA (pdb:1xcg; Derewenda *et al.*, 2004) onto the crystal structure of the SopE–Cdc42 complex (pdb:1gzs; Buchwald *et al.*, 2002). All residues identified by the RhoA titration appear at the interface except for those within the C-terminal helix. In this model the intimate contacts with EspM2 are through the switch regions I and II of RhoA.

Although EspM2^29–196^ is significantly more stable than full length, it has a proposenity to aggregate that can only be alleviated by high salt concentrations and pH values. These factors make EspM2 particularly unsuitable for structural study by either NMR or crystallography. Indeed, despite exhaustive attempts EspM2^29–196^ and full-length EspM2 proved refractory to crystallization (see Appendix S1). Although deuteration of EspM2 was not possible, ∼70% of the backbone resonances could be confidently assigned using standard triple resonance NMR methodology on a ^15^N ^13^C-labelled sample. NMR was then used to monitor the interaction of RhoA with EspM2^29–196^ at a residue-specific level. At 1:1 molar ratio significant changes in ^1^H ^15^N TROSY-HSQC spectra of EspM2 were detectable; and it was possible to determine which assigned EspM2 residues were within intimate proximity of RhoA upon complex formation (Fig. 3A). After the addition of sevenfold molar excess RhoA, peaks corresponding to structured regions of EspM2^29–196^ had broadened to such an extent that many were no longer visible and furthermore, addition of 5 mM GTP to the sample did not result in dissociation of the complex.

The chemical shift index based on NMR data for ^13^C^α^, ^13^C^β^ and ^13^C′ resonances (Wishart *et al.*, 1993) reveals that EspM2 is primarily helical (albeit for an unstructured ∼30 residue region at the N-terminus), which is consistent with secondary structure prediction with PSIPRED (Jones, 1999) (Fig. S1). The helical content and locations are similar to that of the C-terminal domain of SifA whose crystal structure has been recently determined (Fig. S3) (pdb:3cxb; Ohlson *et al.*, 2008). Based on these data we created a sequence alignment between EspM2^29–196^ and SifA, and together with the crystal structure we computed a homology model of EspM2^29–196^ using SWISS-MODEL (Arnold *et al.*, 2006). NMR chemical shift data from RhoA titration was then mapped onto the model of EspM2^29–196^ (Fig. 3B). Furthermore, a model of the complex with RhoA was created by superposing the EspM2^29–196^ homology model and the crystal structure of RhoA (pdb:1xcg; Derewenda *et al.*, 2004) onto the crystal structures of SopE and Cdc42, respectively, within the SopE–Cdc42 complex (pdb:1gzs; Buchwald *et al.*, 2002) (Fig. 3C). In this model of the EspM2–RhoA complex, almost all of the perturbed residues identify from NMR titration experiments lie within the interface and are situated at both of the switch sites of RhoA.

### Site-directed mutagenesis of interface EspM2 residues

**Fig. 4:**
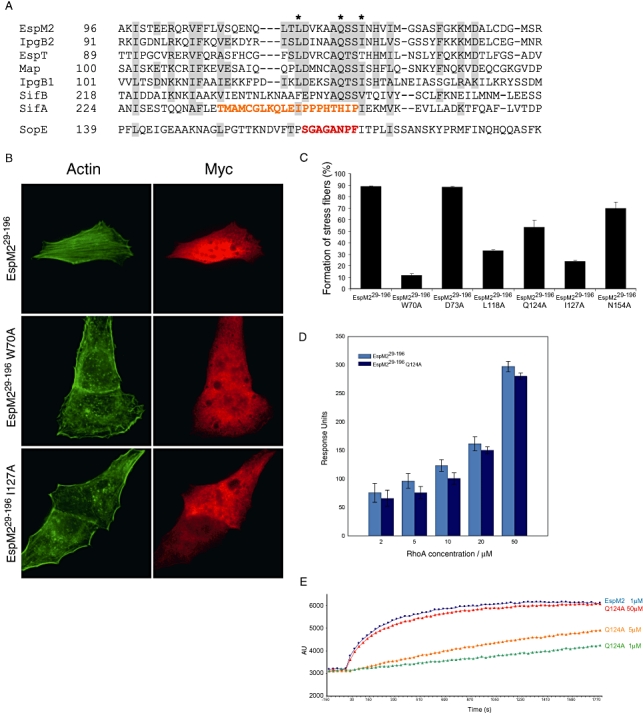
Activity of EspM2^29–196^ mutants. A. Multiple sequence alignment of the putative catalytic loop and flanking regions of the WxxxE proteins: EspM2 of EHEC O157:H7 Sakai, IpgB1 and IpgB2 of *Shigella flexneri*, EspT of *Citrobacter rodentium*, Map of EPEC E2348/69 and SifA and SifB of *S.* Typhimurium. The loop region of SopE was added for comparison, with the catalytic region highlighted. Similar residues are highlighted in grey. A stretch of residues in the putative catalytic loop of SifA that is different from the other WxxxE effectors is highlighted. EspM2 residues selected for mutagenesis are indicated by a star. B. Swiss 3T3 cells were transfected with pRK5 encoding myc-tagged EspM2^29–196^, EspM2^29–196^ W70A and EspM2^29–196^ I127A. Actin was stained with Oregon green phalloidin and the myc tag was detected with monoclonal antibody. C. Quantification of stress fibres formation in cell transfected with different EspM2^29–196^ mutants. Results are displayed as mean ± SEM. D. SPR comparison of RhoA binding to wild-type EspM2 and EspM2 Q124A. No significant difference in binding was detected over a range of RhoA concentrations. Shown is the averaged response of three repeats. E. EspM2^29–196^ Q124A is impaired in loading mant-GTP into RhoA. mant-GTP (0.5 µM) was incubated with 2 µM RhoA in presence of 1 µM EspM2^29–196^ (blue circles) or 1 µM (green triangles), 5 µM (orange triangles) and 50 µM (red triangles) EspM2^29–196^ Q124A.

We introduced alanine substations in EspM2 residues L118A, Q124A and I127A, which are located within the EspM2 loop equivalent to the catalytic domain of SopE, D73, which located within the WxxxE motif and N154, which based on our RhoA-EspM2 model makes a hydrogen bond with the RhoA backbone. EspM2 W70A was used as a control. The EspM2^29–196^ derivatives were expressed ectopically in Swiss 3T3 cells and formation of stress fibres was assessed microscopically (Fig. 4). While the W70A substitution completely abolished function, Q124A substitution attenuated stress fibre formation (seen in 35% of transfected cells compared with cells transfected with wild-type EspM2) while L118A and I127A reduced stress fibre formation by 56% and 65% respectively (Fig. 4). N154A only had a minor effect on stress fibres formation by EspM2 while no effect was detected for D73A (Fig. 4). We tested representative EspM2 mutants delivered form E2348/69 by infection. Consistent with the transfection data W70A and I127A did not trigger stress fibres while D73A, N154A and Q124A triggered stress fibres at levels comparable to the wild-type EspM2 (data not shown).

We next cloned *espM2* W70A, L118A, Q124A and I127A into pET28a for expression as 6His-tagged EspM2^29–196^. Despite repeated attempts we were unable to purified EspM2 W70A and I127A, while the yield of EspM2 L118A was very low. These results suggest that these amino acids play an important structural role, which is consistent with the 3D model (Fig. 3). The Q124A mutant, purified at the same efficiency as the wild-type EspM2, was used in SPR and GEF assays. No significant difference in binding to RhoA was observed between wild-type EspM2 and EspM2 Q124A (Fig. 4). Importantly, EspM2 Q124A was attenuated in its ability to induce loading of GTP into RhoA as 50 µM protein was needed to achieve GTP loading equivalent to that induced by 1 µM wild-type EspM2 (Fig. 4).

## Discussion

When first described the WxxxE effectors were thought to be molecular mimics of Rho GTPases (Alto *et al.*, 2006). Recent data have shown that IpgB1 activates the Rac1 GEF complex ELMO/Dock180 (Handa *et al.*, 2007), and Map, EspT and EspM activate Rho GTPases (Arbeloa *et al.*, 2008; Berger *et al.*, 2009; Bulgin *et al.*, 2009) by an unknown mechanism. In this study we have investigated the mechanism by which EspM2 activates RhoA.

Using surface plasmon resonance we found that EspM2^29–196^ forms a stable complex with nucleotide-free RhoA. The dissociation of the EspM2–RhoA complex was very slow and as a consequence we were unable to measure the dissociation rate constant. We found that the affinity of EspM2^29–196^ to RhoA in presence of GDP or GTP was lower than in absence of nucleotide, which is similar to eukaryotic GEFs that also exhibit higher affinity and form stable complexes with the nucleotide-free Rho GTPases.

The initial step in nucleotide exchange is the formation of a low-affinity complex between the GEF and the GDP-bound Rho GTPase via recognition of the Switch I and II regions (Klebe *et al.*, 1995), which favours GDP and Mg^2+^ release. This is then rapidly converted into a high-affinity GEF–GTPase binary complex (Lai *et al.*, 1993); loading with free GTP leads to dissociation of the GEF and formation of a high-affinity Rho GTPase–GTP complex, which binds subsequently downstream effectors (Milburn *et al.*, 1990). To test if EspM2 has a GEF activity we incubated RhoA with mant-GTP and increasing concentrations of EspM2^29–196^. We found that EspM2^29–196^ induced loading of GTP into RhoA in a concentration-dependent manner. This activity was specific for RhoA as EspM2^29–196^ induced weak nucleotide exchange in Cdc42, while no nucleotide exchange was seen for Rac1 and the distant GTPase H-Ras. Interestingly, the *Salmonella* WxxxE effector SifA, which also binds RhoA, did not exhibit a detectable GEF activity. It is not currently known why binding to RhoA does not lead to nucleotide exchange or if SifA can induce nucleotide exchange in other small GTPases (e.g. Rab).

SopE from *Salmonella* was the first T3SS effector described as a GEF (Hardt *et al.*, 1998). SopE activates Cdc42 and Rac1 leading to formation of membrane ruffles and bacterial invasion. The crystal structure of the SopE–Cdc42 complex illuminated the mechanism by which SopE functions as GEF. SopE is composed of six α-helices arranged in two three-helix bundles forming a V-shape. The junction connecting the two arms consists on small β sheet followed by a loop consisting of the GAGA motif, which is proposed to be the catalytic loop of SopE. Insertion of the GAGA motif between the switch regions of Cdc42 induces a push and pull type movement and release of GDP. Although SopE does not share sequence or structural similarity with eukaryotic GEFs, they induce similar conformational changes in the Rho GTPases. Recently, the crystal structure of SifA in complex with SKIP was solved (Ohlson *et al.*, 2008). While the N-terminal SifA domain binds SKIP, the C-terminal domain, which shares no sequence similarity with SopE and harbours the WxxxE motif, adopts a SopE-like fold. NMR analysis of EspM2^29–196^ combined with homology modelling revealed that it likely contains six to seven α-helices arranged in a V-shape structure similar to SopE and SifA and a loop connecting the two arms (Fig. S3). Interestingly, the sequence of this putative catalytic loop is conserved between EspM2^29–196^ and the other WxxxE effectors, but not SifA (Fig. 4A), which might provide an explanation as for why SifA cannot induce nucleotide exchange in RhoA.

The tryptophan and glutamic acid of the WxxxE motif have been shown to be essential as replacement of W or E by alanine completely abolished the effectors' function (Alto *et al.*, 2006; Arbeloa *et al.*, 2008). However, we recently found that conservative substitutions of the W and E residues in EspM2 had little effect on stress fibre formation, suggesting that they have a structural role. In our model the W70 and E74 residues in EspM2 are positioned around the junction of the two three-helix bundles. Consistently, the role proposed for these residues in SifA was to maintain the conformation of the putative catalytic loop through hydrophobic contacts with surrounding residues. Among these residues I258, the equivalent of I127 in EspM2, forms hydrophobic contacts with the tryptophan and hydrophobic contacts and a hydrogen bond with the glutamic acid (Ohlson *et al.*, 2008). We observed that alanine substitutions of W70 and I127 of EspM2 abolished almost completely its ability to induce formation of stress fibres. Despite repeated attempts we were unable to purify His-tagged EspM2 W70 and I127 suggesting that these residues play a role in maintaining the global structure, which is consistent with the 3D model of EspM2.

We use NMR to investigate how formation of a complex with RhoA affects the conformation of EspM2^29–196^. Titrating RhoA into ^15^N ^13^C-labelled EspM2^29–196^ revealed specific protein interaction with most of the shifted peaks being located either in the penultimate helix or in the putative catalytic loop. Other than I127, substituting the putative EspM catalytic loop residue L118 by alanine also had a substantial effect on stress fibres formation; however, the recombinant protein was highly unstable.

While this project was reaching conclusion, Huang *et al.* (2009) published the crystal structure of the Map–Cdc42 complex and showed that Map induces guanine nucleotide exchange in Cdc42 while IpgB2 and IpgB1 induce nucleotide exchange in RhoA and Rac1 respectively. In this study it was shown that Map Q128, located within the catalytic loop, makes a hydrogen bond with Cdc42 Phe37, which fortifies the interaction between these two proteins. Moreover Map Q128Y does not bind to, or induce nucleotide exchange in, Cdc42. Interestingly, while not affecting the interaction of EspM2 with RhoA, EspM2 Q124A (equivalent to Map Q128) was attenuated in stress fibre formation by transfection and RhoA GEF activity; 50 µM EspM2 Q124A was needed to achieve the same GEF activity as 1 µM wild-type EspM2. Importantly, while mutations affecting protein stability were inactive when delivered either by transfection or by infection, we did not detect a significant effect on stress fibre formation when EspM2 Q124A (or D73A and N154A) were delivered by infection. This suggests that upon translocation the local effector concentration is high enough to induce nucleotide exchange, compared with ectopic expression which leads to global cytosolic distribution, low effector concentration and hence an attenuated stress fibre formation.

The high-affinity complexes formed between GEFs and their cognate Rho GTPases are quickly dissociated upon addition of GTP or GDP. Unexpectedly we found that the EspM2^29–196^–RhoA and SifA–RhoA complexes did not dissociate in the presence of exogenous nucleotides. This result suggests that EspM2 could activate RhoA by a unique mechanism, involving constitutive activation, which allows recruitment of ROCK to the EspM2–RhoA complex. Supporting this hypothesis is our finding that upon infection of Swiss 3T3 cells with EPEC overexpressing EspM2 the induced stress fibres were stable for at least 5 h after the adherent bacteria were killed by gentamicin (not shown). Moreover, Alto *et al.* (2006) have shown that IpgB2 is co-immunoprecipitated with ROCK, possibly via mutual interaction with RhoA.

In conclusion, our work shows that EspM2 is a RhoA GEF. Importantly, a large screen of over 900 clinical EPEC and EHEC isolates revealed that *espM* is found in *c*. 50% of the strains (Arbeloa *et al.*, 2009). It is now well documented that while 21 core effector genes are conserved in all EPEC and EHEC strains, the distribution of the non-conserved, accessory, effector genes varies from strain to strains (Iguchi *et al.*, 2009). This suggests that EPEC and EHEC strains can employ different infection strategies. Although the role of EspM2 during infection is not yet known, a recent study by Simovitch *et al.* (2009) showed that it is involved in delocalization of the thigh junctions (TJ). Importantly, the TJ alterations induced by EspM2 did not interfere with their functionality; on the contrary, increased TER values were observed upon EspM2 expression. These results suggest that EspM2 might play a role in maintaining TJ during infection. Moreover, EspM2 might modulate additional cellular pathways, as IpgB2 was recently implicated in activation of NF-kB in a RhoA-ROCK-dependent manner (Fukazawa *et al.*, 2008). Further studies are needed in order to determine the role played by EspM *in vivo*.

## Experimental procedures

### Bacterial strains and cell culture

Bacteria were grown from single colonies in Luria–Bertani (LB) broth in a shaking incubator at 37°C or maintained on LB plates. Culture medium was supplemented with ampicillin (100 µg ml^−1^) or kanamycin (25 µg ml^−1^) as appropriate.

Swiss 3T3 cells were maintained in DMEM with 4500 mg ml^−1^ glucose and supplemented with 10% fetal calf serum (Gibco) and 4 mM Glutamax (Gibco).

### Plasmids and molecular techniques

Plasmids used in this study are listed in Table S1 in *Supporting information*; primers are listed in Table S2. *espM2* and *espM2^29–196^* were amplified by PCR using genomic EHEC O157:H7 strain Sakai DNA as template and cloned into pET28 with non-cleavable N-terminal 6His tag or into the mammalian expression vector pRK5 with an N-terminal myc tag. *sifA* was amplified by PCR using genomic *S.* Typhimurium SL1344 DNA as template and cloned into pET28 with an N-terminal 6His tag. All constructs were verified by DNA sequencing.

The vector pMW172-His expressing RhoA or Rac1 fused to a 6His Tag was a gift from Michael Way.

### Site-directed mutagenesis

Site-directed mutagenesis was carried out using a Quickchange II kit (Stratagene) according to the manufacturer's instructions. Primers were designed using the Quickchange mutagenic primer design program (Stratagene). Plasmids pRK5:*espM2^29–196^*, pET28:*espM2^29–196^* and pSA10:*espM2* were used as template for the mutagenic reactions. All constructs were verified by DNA sequencing.

### Preparation of recombinant proteins

*Escherichia coli* B834 containing pET28a (NdeI/BamHI inserted *espM2^29–196^* or *SifA*) were grown at 310 K in LB broth containing 25 mg ml^−1^ kanamycin until an *A*_600_ of 0.6 was reached. Protein overexpression was induced by the addition of 1.0 mM IPTG. Cells were harvested after incubation at 293 K overnight (4500 *g*, 20 min, 277 K). Cell pellets were re-suspended in lysis buffer (20 mM Tris pH 8, 500 mM NaCl, 1 mM DTT), complete EDTA-free protease inhibitor cocktail (Roche) and homogenized. After centrifugation (14 000 *g*, 30 min, 277 K), the soluble fraction was applied to a 5 ml His trap FF Column (GE Healthcare) in a gradient of 0–300 mM imidazole. EspM2 fractions were pooled and purified using a Superdex 75 26/60 equilibrated in 20 mM Tris pH 8, 500 mM NaCl, 10 mM DTT, high salt due to the instability of the protein. Protein was Liquid Nitrogen flash cooled and frozen at 193 K on the same day, after experiencing protein precipitation in samples kept at 277 K over 2–3 days.

Preparation of His-RhoA and His-Rac-1 was identical with the following exceptions: expression was constitutive; cells were harvested after incubation at 310 K overnight. Lysis buffer was 20 mM Tris, 500 mM NaCl, 20 mM imidazole, 5 mM MgCl_2_. Size exclusion buffer was 20 mM Tris, 200 mM NaCl, 3 mM MgCl_2_, 1 mM DTT.

### Surface plasmon resonance

Experiments were carried out using a BIAcore™ 2000 System (Biacore AB, Sweden) at 20°C. EspM2^29–196^ was coupled to a CM5 sensor chip leading to a rise of 2000 Response Units using standard amine coupling protocols (BIAcore Amine Coupling Kit BR-1000-50). Hepes-buffered saline containing 5 mM MgCl_2_ was flowed throughout the experiment at 50 µl min^−1^. Samples of RhoA/Rac-1 were injected (in the same buffer) for 180 s duration over the EspM2^29–196^ channel and over a RhoA control channel. Dissociation was followed for 1000 s from the end of injection after which regeneration of the surface was carried out with 25 mM NaOH. No loss of activity was seen after regeneration. Experiments (repeated three times) were carried out with (i) varying concentrations of RhoA (*ε* = 18 825 M^−1^ cm^−1^) and Rac-1 (*ε* = 23 295 M^−1^ cm^−1^) (concentration determined by Nanodrop Spectrophotometer, 0.005–50 µM), and with (ii) fixed 4 µM RhoA but varying concentrations of GDP/GTP (0.5–8 µM) added to the buffer. RhoA control channel subtracted signals are presented (BIAEvaluation software). The same conditions were used to analyse the SifA–RhoA interaction.

### Guanine nucleotide exchange analysis

*In vitro* GEF activity assay was carried out using RhoGEF exchange assay biochem kit (Cytoskeleton Denver) according to the manufacturer's instructions. Briefly, exchange reaction assay mixtures contained 20 mM Tris pH 7.5, 50 mM NaCl, 10 mM MgCl_2_, 50 µg ml^−1^ bovine serum albumin (BSA), 0.75 µM *N*-methylanthraniloyl(mant)-GTP, and 2 µM Rac1-His, Cdc42-His, H-Ras-His or RhoA-His GTPase. Fluorescence spectroscopic analysis of mant-GTP was carried out using Fluostar Optima spectrometer. The fluorescence measurements were taken every 30 s with excitation and emission wavelengths of 360 nm and 440 nm respectively. After five readings (150 s), purified EspM2^29–196^ was added and the relative mant fluorescence was monitored every 30 s for a total time of 30 min. Experiments were performed in triplicate.

### NMR sample preparation and backbone assignment of EspM2^29–196^

Uniformly ^15^N ^13^C-labelled EspM2^29–196^ was expressed in ^15^N ^13^C-labelled rich media (Cambridge isotopes). After purification as described above the sample was dialysed against NMR buffer [50 mM NaPO_4_ pH 7.5, 150 mM NaCl, 5 mM DTT, 10% (v/v) D_2_O] and concentrated to 0.9 mM. Backbone assignment for ∼65% of EspM2^29–196^ was achieved using standard double- and triple-resonance assignment experiments (Sattler *et al.*, 1999) at 295 K.

### NMR titration of RhoA against EspM2^29–196^

^15^N ^13^C-labelled EspM2^29–196^ was mixed with unlabelled His-RhoA (dialysed in NMR buffer) ranging from 0x to 7x molar excess and ^1^H ^15^N TROSY-HSQC experiments were performed at each increment at 298 K. GTP (5 mM) was added to the sample of EspM2^29–196^ saturated with RhoA and a final ^1^H ^15^N TROSY-HSQC was carried out.

### Transfection

Swiss 3T3 cells were transfected with pRK5 encoding EspM2, EspM2^29–196^ and derivatives fused to a myc tag by lipofectamine 2000 (Invitrogen), according to the manufacturer's recommendations. The cells were incubated at 37°C in a humidified incubator with 5% CO_2_ for 19 h, washed twice in PBS before having their media replaced with DMEM as described previously (Arbeloa *et al.*, 2008).

### Infection of Swiss 3T3

Forty-eight hours prior to infection cells were seeded onto glass coverslips at a density of 5 × 10^5^ cells per well and maintained in DMEM supplemented with 10% FCS at 37°C in 5% CO_2_. Three hours before infection the cells were washed three times with PBS, the media replaced with fresh DMEM without FCS and 500 µl of primed bacteria were added to each well and infections were carried out for 90 min at 37°C in 5% CO_2_.

### Immunofluorescence staining and microscopy

Swiss 3T3 cells on coverslips were washed three times in PBS and fixed with 3% paraformaldehyde for 20 min before washing three more times with PBS. For immunostaining, the cells were permeabilized for 4 min in PBS 0.5% Triton X-100, washed three times with PBS and quenched for 30 min with 50 mM NH_4_Cl. The coverslips were then blocked for 1 h with 10% donkey serum (Jackson laboratories) before incubation with primary and secondary antibodies. The primary antibody mouse anti-myc (Millipore) was used at a dilution of 1:500. Coverslips were incubated with the primary antibody for 1 h, washed three times in PBS and incubated with the secondary antibody for 1 h. Donkey anti-mouse IgG conjugated to a Cy3 fluorophore (Jackson laboratories) was used at a 1:200. Actin was stained using Oregon Green phalloidin (Invitrogen) at a 1:100 dilution. All dilutions were in 10% donkey serum. Coverslips were mounted on slides using ProLong Gold antifade reagent (Invitrogen) and visualized by Zeiss Axioimager immunofluorescence microscope using the following excitation wavelengths: Cy3 – 550 nm, Cy5 – 650 nm and Oregon Green – 488 nm. All images were analysed using the Axiovision Rel 4.5 software.

## Supporting information

**Appendix S1.** Supplementary results.

**Fig. S1.** ^1^H ^15^N HSQC spectra of EspM2 constructs at 295 K.

A. Full-length EspM2 where the overlapping peaks within the centre of the spectra are due to resonances from highly flexible regions of the protein.

B. The much increased resolution of the spectra of EspM2^29–196^ shows that this truncated form is a compact structure with an appropriate number of amide peaks.

**Fig. S2.** Activity of EspM2^29–196^.

A. Serum-starved Swiss 3T3 cells were mock transfected or transfected with the mammalian expression vector pRK5 encoding myc-tagged EspM2 and EspM2^29–196^ for 19 h. Actin was stained with Oregon green phalloidin and the myc tag was detected with monoclonal antibody. Transfection of EspM2 and EspM2^29–196^ induces the formation of parallel stress fibres to the same extent.

B. Quantification of stress fibres on Swiss 3T3 after 19 h transfection with EspM2 and EspM2^29–196^. Fifty cells were counted in duplicate in three independent experiments. Results are displayed as mean ± SEM.

**Fig. S3.** Secondary structure of EspM2^29–196^. From the NMR chemical shift index (CSI) (Wishart *et al.*, 1993), predictions with PSIPRED (Jones, 1999) and a model based on the crystal structure of the C-terminal domain of SifA (pdb:3cxb; Ohlson *et al.*, 2008) EspM2^29–196^ most likely contains between six and seven helices and two short N-terminal helices. Helices are shown as red rectangles, sheets as blue arrows, coils and loops are grey lines while regions in the CSI which have not been assigned are left blank.

**Table S1.** List of plasmids.

**Table S2.** List of primers.
