# Supplementary material for: EspM2 is a RhoA guanine nucleotide exchange factor
Source: Cell Microbiol. 2010 Feb 5;12(5):654–64. doi: 10.1111/j.1462-5822.2009.01423.x (PMC2871174; doi:10.1111/j.1462-5822.2009.01423.x)
Supplement: Supplementary file 1 [file cmi0012-0654-SD1.pdf]

# SUPPLEMENTARY DATA

## SUPPLEMENTARY RESULTS

*EspM2 is highly unstable*

Although full length EspM2 expresses extremely well with yields in excess of 40 mg/L of culture, it is highly unstable and in order to maintain soluble homogenous material it was essential that all buffers were supplemented with at least 500 mM NaCl and experiments performed for long durations required 1M NaCl. Furthermore, whilst EspM2 is stable for a number of weeks at 277 K, it precipitates after only a few hours above 295 K and in all, these conditions are by no means ideal for structural studies by NMR. Once a $^{1}H$ $^{15}N$ HSQC spectrum had been acquired it became evident that a proportion of EspM2 was unstructured (Supplementary Fig. 1A) and treatment with trypsin yielded a product which was much more amenable for structural studies (EspM2$^{29\text{-}196}$). A $^{1}H$ $^{15}N$ HSQC spectrum of EspM2$^{29\text{-}196}$ showed that it is a compact protein and only 150 mM NaCl is required to maintain homogeneity, but at pH below 7.5 and temperatures in excess of 295 K precipitation was evident. It is also worth noting that although two independent groups have tried to crystallise EspM2, the truncated form and complexes with RhoA, it has not been possible.

Table S1. List of plasmids

| Name | Description | Reference |
|---|---|---|
| pICC453 | pET28::*espM2*$^{29\text{-}196}$ | This work |
| | pMW172-*His-RhoA* F25N | (Valderrama *et al.*, 2006) |
| | pMW172-*His-Rac1* | (Valderrama *et al.*, 2006) |
| pICC454 | pET28::*sifA* | This work |
| pICC455 | pRK5::*myc-espM2* | (Arbeloa *et al.*, 2008) |
| pICC456 | pRK5::*myc-espM2*$^{29\text{-}196}$ | This work |
| pICC457 | pRK5::*myc-espM2*$^{29\text{-}196}$ W70A | This work |
| pICC458 | pRK5::*myc-espM2*$^{29\text{-}196}$ L118A | This work |
| pICC459 | pRK5::*myc-espM2*$^{29\text{-}196}$ Q124A | This work |
| pICC460 | pRK5::*myc-espM2*$^{29\text{-}196}$ I127A | This work |
| pICC494 | pRK5::*myc-espM2*$^{29\text{-}196}$ D73A | This work |
| pICC495 | pRK5::*myc-espM2*$^{29\text{-}196}$ N154A | This work |
| pICC496 | pET28::*espM2*$^{29\text{-}196}$ W70A | This work |
| pICC497 | pET28::*espM2*$^{29\text{-}196}$ L118A | This work |
| pICC498 | pET28::*espM2*$^{29\text{-}196}$ Q124A | This work |
| pICC499 | pET28::*espM2*$^{29\text{-}196}$ I127A | This work |
| pICC398 | pKK:*espM2*-HA | (Arbeloa *et al.*, 2008) |
| pICC404 | pKK:*espM2*-HA W70A | (Arbeloa *et al.*, 2008) |
| pICC500 | pKK:*espM2*-HA D73A | This work |
| pICC501 | pKK:*espM2*-HA Q124A | This work |
| pICC502 | pKK:*espM2*-HA I127A | This work |
| pICC503 | pKK:*espM2*-HA N154A | This work |

Table S2. List of primers

| Primer | Sequence |
| --- | --- |
| espM2(29-196)-His F | 5'-catgCCATGGgaCATCATCATCATCATCACggaggaGGGAAGAATGA[illegible] |
| espM2(29-196)-His R | 5'-aaGAATTCTCATCCCTGTATAGCACGCA-3' |
| sifA-His F | 5'-ggaattcCATATGATGCCGATTACTATAGGGA-3' |
| sifA-His R | 5'-aaagaattCTTATAAAAAACAACATAAACAGCCGC-3' |
| espM2(29-196)-myc F | 5'-tttGAATTCaGGGAAGAATGATGAGGTCGT-3' |
| espM2(29-196)-myc R | 5'-ccaatgcattggttCTGCAGTCATCCCTGTATAGCACGCA-3' |
| espM2 W70A F | 5'-gcagagtactaaagatattgatgaggcgataaaagatgaacggatagtatat-3' |
| espM2 W70A R | 5'-atatactatccgttcatcttttatcgcctcatcaatatctttagtactctgc-3' |
| espM2 L118A F | 5'-ccaggagaatcaactaaccgctgatgttaaggcagcacaa-3' |
| espM2 L118A R | 5'-ttgtgctgccttaacatcagcggttagttgattctcctgg |
| espM2 Q124A F | 5'-actaacccttgatgttaaggcagcagcaagttctattaatcatgtaataatg-3' |
| espM2 Q124A R | 5'-cattattacatgattaatagaacttgctgctgccttaacatcaagggttagt-3' |
| espM2 I127A F | 5'-gatgttaaggcagcacaaagttctgctaatcatgtaataatggggagtgc-3' |
| espM2 I127A R | 5'-gcactccccattattacatgattagcagaactttgtgctgccttaacatc-3' |
| espM2 D73A F | 5'-taaagatattgatgagtggataaaagctgaacggatagtatatccc-3' |
| espM2 D73A R | 5'-gggatatactatccgttcagcttttatccactcatcaatatcttta-3' |
| espM2 N154A F | 5'-ttctattaatcatgtaataatggcgagtgcttcttttggcaagaaaa-3' |
| espM2 N154 R | 5'-ttttcttgccaaaagaagcactcgccattattacatgattaatagaa-3' |

**Supplementary Figure 1: $^{1}H$ $^{15}N$ HSQC spectra of EspM2 constructs at 295 K.** A. Full length EspM2 where the overlapping peaks within the centre of the spectra are due to resonances from highly flexible regions of the protein. B. The much increased resolution of the spectra of EspM2$^{29-196}$ shows that this truncated form is a compact structure with an appropriate number of amide peaks.

**Supplementary Figure 2: Activity of EspM2$^{29-196}$.** A. Serum-starved Swiss 3T3 cells were mock transfected or transfected with the mammalian expression vector pRK5 encoding myc-tagged EspM2 and EspM2$^{29-196}$ for 19 h. Actin was stained with Oregon green phalloidin and the myc tag was detected with monoclonal antibody. Transfection of EspM2 and EspM2$^{29-196}$ induces the formation of parallel stress fibers to the same extent. B. Quantification of stress fibers on Swiss 3T3 after 19h transfection with EspM2 and EspM2$^{29-196}$. Fifty cells were counted in duplicate in three independent experiments. Results are displayed as mean ± SEM.

**Supplementary Figure 3: Secondary structure of EspM2$^{29-196}$**. From the NMR chemical shift index (CSI) (Wishart *et al.*, 1993), predictions with PSIPRED (Jones, 1999) and a model based on the crystal structure of the C-terminal domain of SifA (pdb:3cxb;(Ohlson *et al.*, 2008)) EspM2$^{29-196}$ most likely contains between 6-7 helices and 2 short N-terminal helices. Helices are shown as red rectangles, sheets as blue arrows, coils and loops are grey lines whilst regions in the CSI which have not been assigned are left blank.

## References

Arbeloa, A., Bulgin, R.R., MacKenzie, G., Shaw, R.K., Pallen, M.J., Crepin, V.F., *et al* (2008) Subversion of actin dynamics by EspM effectors of attaching and effacing bacterial pathogens. *Cell Microbiol* **10**: 1429-1441.

Jones, D.T. (1999) Protein secondary structure prediction based on position-specific scoring matrices. *J Mol Biol* **292**: 195-202.

Ohlson, M.B., Huang, Z., Alto, N.M., Blanc, M.P., Dixon, J.E., Chai, J. and Miller, S.I. (2008) Structure and function of Salmonella SifA indicate that its interactions with SKIP, SseJ, and RhoA family GTPases induce endosomal tubulation. *Cell Host Microbe* **4**: 434-446.

Valderrama, F., Cordeiro, J.V., Schleich, S., Frischknecht, F. and Way, M. (2006) Vaccinia virus-induced cell motility requires F11L-mediated inhibition of RhoA signaling. *Science* **311**: 377-381.

Wishart, D.S., Sykes, B.D. and Richards, F.M. (1993) Improved synthetic methods for the selective deuteration of aromatic amino acids: applications of selective protonation towards the identification of protein folding intermediates through nuclear magnetic resonance. *Biochim Biophys Acta* **1164**: 36-46.

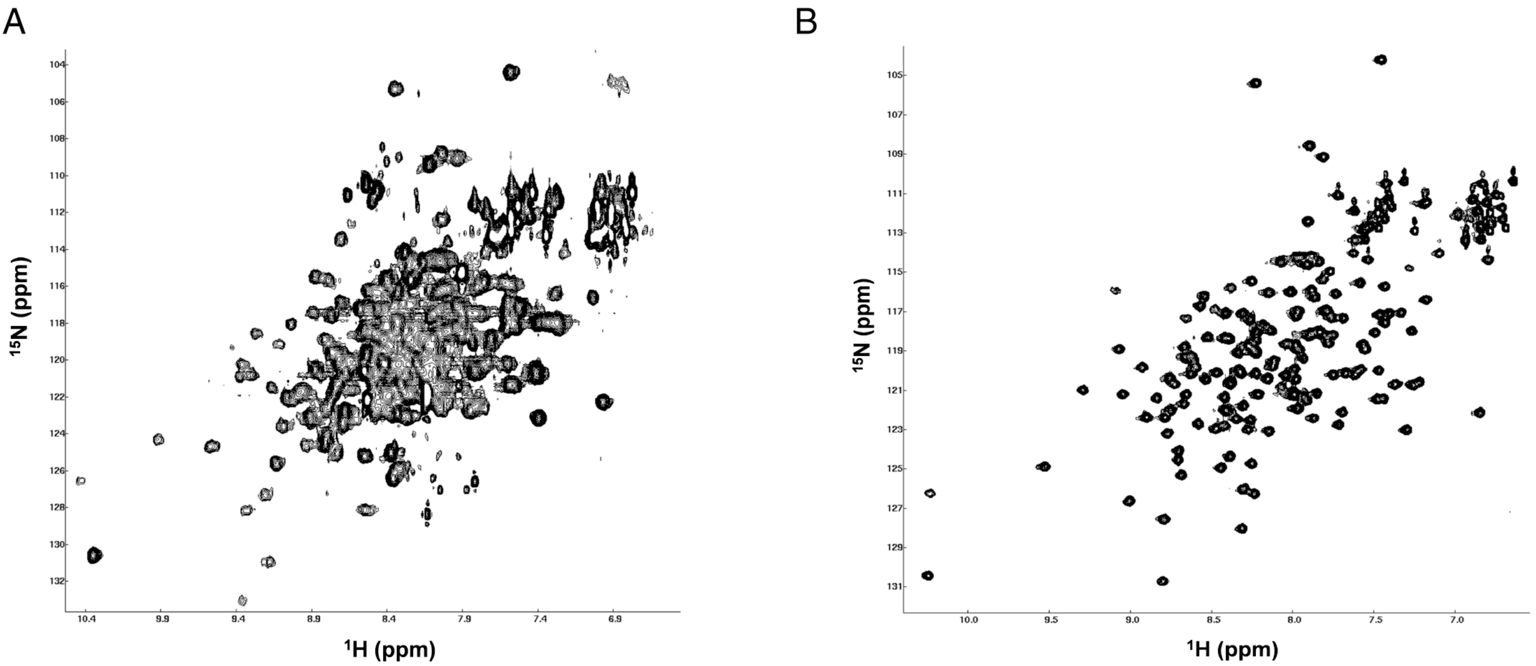


Supplementary Figure 1

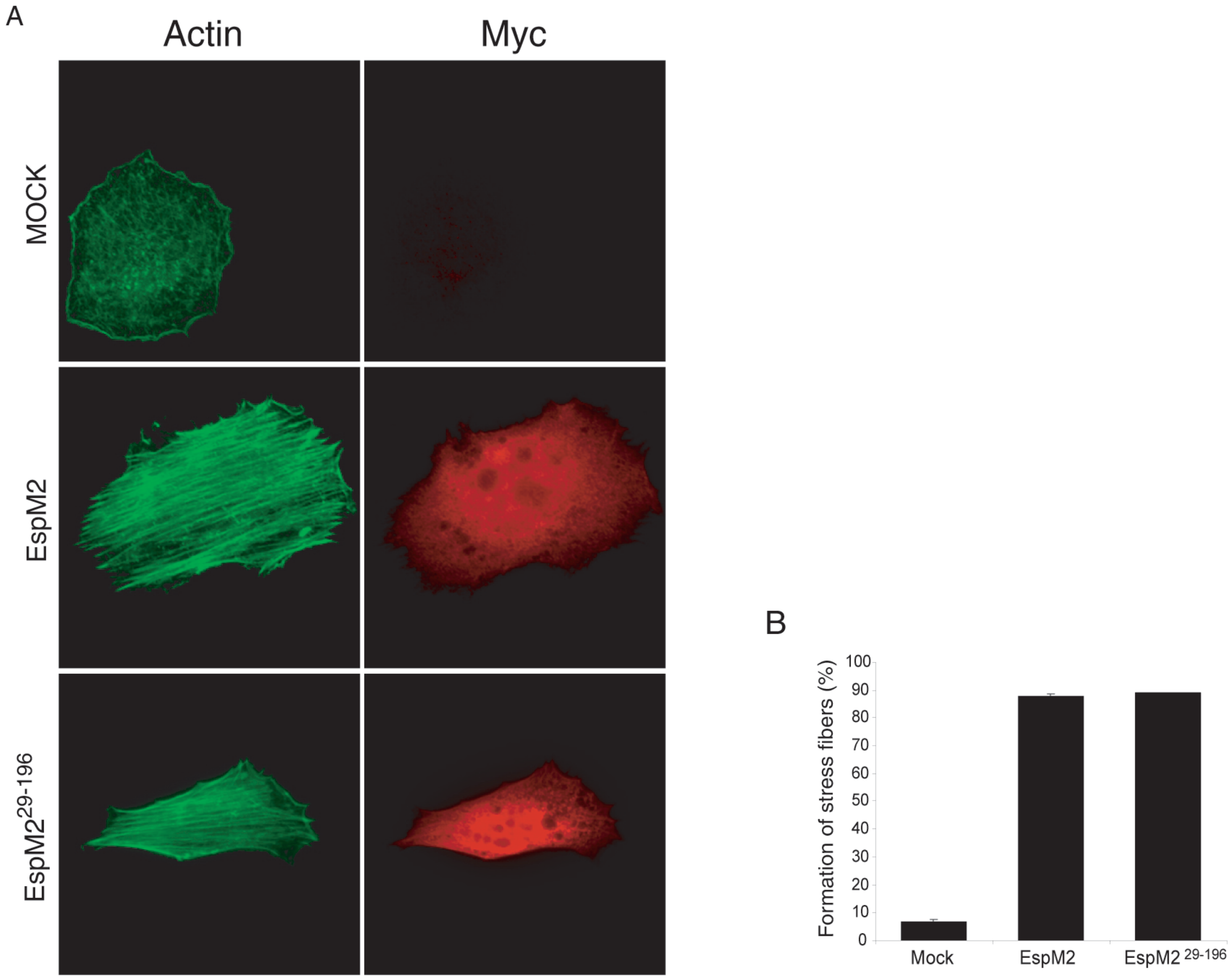


Supplementary Figure 2

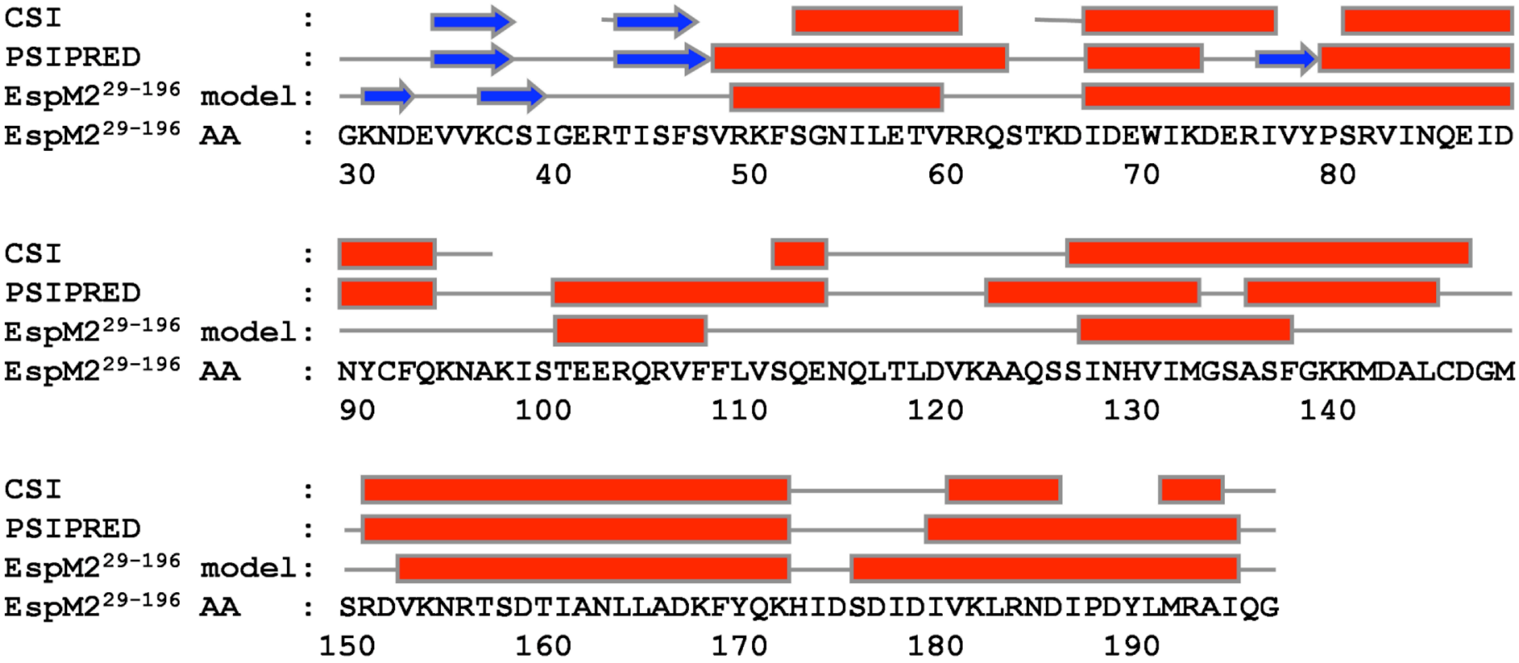


Supplementary Figure 3
